# Ocular *Chlamydia trachomatis* infection and infectious load among pre-school aged children within trachoma hyperendemic districts receiving the SAFE strategy, Amhara region, Ethiopia

**DOI:** 10.1371/journal.pntd.0008226

**Published:** 2020-05-18

**Authors:** Scott D. Nash, Ambahun Chernet, Jeanne Moncada, Aisha E. P. Stewart, Tigist Astale, Eshetu Sata, Mulat Zerihun, Demelash Gessese, Berhanu Melak, Gedefaw Ayenew, Zebene Ayele, Melsew Chanyalew, Thomas M. Lietman, E. Kelly Callahan, Julius Schachter, Zerihun Tadesse

**Affiliations:** 1 Trachoma Control Program, The Carter Center, Atlanta, United States of America; 2 Trachoma Control Program, The Carter Center, Addis Ababa, Ethiopia; 3 Department of Laboratory Medicine, University of California, San Francisco, United States of America; 4 Health Promotion and Disease Prevention Core Process, Amhara Regional Health Bureau, Bahir Dar, Ethiopia; 5 Francis I. Proctor Foundation, University of California, San Francisco, United States of America; RTI International, UNITED REPUBLIC OF TANZANIA

## Abstract

**Background:**

After approximately 5 years of SAFE (surgery, antibiotics, facial cleanliness, environmental improvement) interventions for trachoma, hyperendemic (trachomatous inflammation-follicular (TF) ≥30%) districts remained in Amhara, Ethiopia. This study’s aim was to characterize the epidemiology of *Chlamydia trachomatis (Ct)* infection and load among pre-school aged children living under the SAFE strategy.

**Methods:**

Conjunctival swabs from a population-based sample of children aged 1–5 years collected between 2011 and 2015 were assayed to provide *Ct* infection data from 4 endemic zones (comprised of 58 districts). *Ct* load was determined using a calibration curve. Children were graded for TF and trachomatous inflammation-intense (TI).

**Results:**

7,441 children were swabbed in 4 zones. TF and TI prevalence were 39.9% (95% confidence Interval [CI]: 37.5%, 42.4%), and 9.2% (95% CI: 8.1%, 10.3%) respectively. *Ct* infection prevalence was 6.0% (95% CI: 5.0%, 7.2%). Infection was highest among children aged 2 to 4 years (6.6%-7.0%). Approximately 10% of infection occurred among children aged 1 year. *Ct* load decreased with age (P = 0.002), with the highest loads observed in children aged 1 year (P = 0.01) vs. aged 5 years. Participants with TF (P = 0.20) and TI (P<0.01) had loads greater than individuals without active trachoma.

**Conclusions:**

In this hyperendemic setting, it appears that the youngest children may contribute in meaningful ways towards persistent active trachoma.

## Author summary

Current World Health Organization recommendations for the treatment of trachoma state that hyperendemic districts, districts with a 30% or greater prevalence of the clinical sign trachomatous inflammation-follicular, should receive 5 years of annual mass drug administration with antibiotic to target *Chlamydia trachomatis* as part of the surgery, antibiotic, facial cleanliness, and environmental improvement (SAFE) strategy. After approximately 5 years of SAFE in Amhara region, Ethiopia, however, many districts remained hyperendemic as measured by observed clinical signs. Given the persistent nature of trachoma in Amhara, a better understanding of the nature of actual *Chlamydia trachomatis* infection, including the distribution of infectious load within children, would be helpful for Amhara and for programs serving other hyperendemic regions. From 2011 to 2015 nearly 7,500 ocular swabs from 4 endemic zones were collected to monitor *Chlamydia trachomatis* following 5 years of SAFE among children aged 1 to 5 years, the group most at risk for infection. It was determined that pre-school age children still harbored considerable infection. The youngest children had the highest infection prevalence and highest infectious load burden, and therefore likely contributed in meaningful ways towards the persistent active trachoma observed in some districts. Treatment regimens focused on the youngest children or children harboring the highest infectious loads should be explored to help countries experiencing persistent trachoma reach elimination as a public health problem faster.

## Introduction

Trachoma is a blinding disease caused by the obligate intracellular bacterium *Chlamydia trachomatis (Ct)*. The World Health Organization (WHO) recommends the SAFE (Surgery, Antibiotics, Facial cleanliness, and Environmental improvement) strategy for the elimination of trachoma as a public health problem [1]. This strategy calls for annual mass drug administration (MDA) of the antibiotic azithromycin to all individuals aged 6 months and older and the administration of topical tetracycline eye ointment for children aged under 6 months for a minimum of 5 years, if the district (the administrative unit for health care management) prevalence of trachomatous inflammation-follicular (TF) among children aged 1 to 9 years is ≥ 30%.

The global trachoma program has seen considerable success with the SAFE strategy as 9 formerly endemic countries have recently been validated as having eliminated trachoma as a public health problem [2]. In the Amhara region of Ethiopia, despite an average of 5 years of SAFE including annual MDA with azithromycin, trachoma remained hyperendemic in many districts (locally known as woredas) as measured by the indicator TF [3]. It was further demonstrated that considerable *Ct* infection remained in many districts despite the magnitude of SAFE interventions [4].

The methodology for previous *Ct* infection research in Amhara called for pooling ocular swabs to save time and resources [4–6]. Although this allows for estimating prevalence at the district level as well as the zonal level (a collection of districts), it limits the ability to study infection and infectious load among individuals. Higher infectious loads have been shown to be associated with disease severity and with a higher likelihood of infection post MDA [7–12]. Children with high loads could also potentially be sources of reinfection in the community [9]. Given the persistent nature of trachoma in Amhara, a better understanding of individual level infection, including the distribution of infectious load within children, would be helpful for the Amhara program and for programs serving other hyperendemic regions.

Conjunctival swabs were collected from a population-based sample of children aged 1 to 5 years throughout the Amhara region. Swabs from *Ct*-positive pools identified from 4 zones (North Gondar, South Gondar, East Gojam and Waghemra, a total of 58 districts) which remained highly endemic despite the SAFE strategy were assayed individually to provide individual level infection data. The aim of this study was to better characterize the epidemiology of ocular *Ct* infection and infectious load among pre-school aged children born predominantly during the implementation of the SAFE strategy in 1 of the most historically trachoma hyperendemic countries of the world.

## Methods

### Ethical statement

Survey methods were reviewed and approved by the Emory University Institutional Review Board (protocol 079–2006) as well as by the Amhara Regional Health Bureau. Due to the high illiteracy rate among the population, Institutional Review Board approval was obtained for oral consent or assent. Oral consent or assent was obtained and recorded electronically for all individual participants according to the principles of the Declaration of Helsinki.

### Setting

Based on the results of baseline trachoma surveys and the availability of the Zithromax donation program, the Amhara region started to scale up the SAFE strategy in 2007. Because of the size of the region, it took between 2007 and 2010 for this scale up to reach all districts. Between 2007 and 2015, over 124 million doses of antibiotic were distributed to the Amhara region [3]. Administrative coverage, defined as doses of drug distributed divided by the total targeted population, was demonstrated to be high between 2008 and 2015 in Amhara (S1 Fig). Programmatic coverage surveys conducted in 2011 and 2012 found district-level self-reported coverage estimates ranging from 79.5% to 94.4%, supporting this achievement of high coverage within these populations [13].

As has been reported extensively in the literature, the Trachoma Control Program in Amhara also implemented F and E interventions between 2007 and 2015 [3, 14–17]. Briefly, once at scale, the program provided village-based health education on face washing and hygiene to approximately 3,400 villages per year, and school-based health education to approximately 8,000 schools per year. Furthermore, the program has helped to promote latrine construction throughout the region, assisting in the construction of nearly 3.5 million latrines during this time period. Previously published reports have detailed increases in the presence of improved water sources [3, 15] and increases in latrines [3, 15, 16] throughout the region. Despite these improvements, however, the prevalence of latrines in Amhara at the time of these current surveys was 50.2%, and access to a water source within 30 minutes was 66.2% region-wide [3].

For this study we focused on 4 contiguous zones of Amhara, which, ranged in TF prevalence from 28.9% to 60.1% among children aged 1 to 9 years [3]. Most districts (39/58, 67.2%) within these 4 zones had received 5 years (range 5–7 years) of SAFE interventions prior to these surveys.

### Surveys

From 2011 to 2015 population-based trachoma surveys were conducted in all districts of Amhara to assess the impact of approximately 5 years of SAFE interventions. Because the SAFE strategy was scaled up over a period of 5 years (2007–2010), it took 5 years to survey all districts in the region. Sampling methodology for these surveys has been published previously, but briefly, a multi-stage cluster randomized methodology was used, whereby clusters (villages) were selected using a population proportional to estimated size method, and within a cluster, a modified segmentation approach was used to randomly select households [3–5, 18].

All residents aged > 1 year in all selected households were enumerated, and present and consented residents were examined for the 5 WHO simplified signs of trachoma using x2.5 magnification and adequate light, including a flashlight if necessary [19]. Individuals diagnosed with TF and/or trachomatous inflammation-intense (TI) were offered treatment with 1% tetracycline eye ointment to be used twice daily for 6 weeks according to current WHO guidelines. Every-other cluster was chosen for swab collection prior to surveying a district, and during the house to house survey, the first 25 children aged 1 to 5 years with parental consent per cluster of 30 to 40 households were swabbed for the presence of infection. If more than 1 child in a household was in this age-range, survey software randomly selected 1 child to be swabbed.

### Training

Prior to each survey round, approximately twice per year, grader trainees participated in a standardized training to be certified to grade trachoma [3, 4]. Grader trainees were taken from a pool of trained integrated eye care workers. These trainings were typically 7 days in length and consisted of classroom and field-based practice. Trainees were required to pass a field-reliability exam whereby 50 conjunctivae were graded from children aged < 10 years. Trainees who scored a kappa ≥ 0.7 for the TF sign when compared to the consensus grade of 3 expert trachoma graders moved into the field as graders on survey teams. Graders who participated in multiple survey rounds were required to pass the field reliability exam prior to each survey round to protect against grading drift.

### Conjunctival swab collection and laboratory procedures

Conjunctival swabbing as part of these surveys has been described previously [4, 5]. Briefly, gloved graders swabbed the upper tarsal conjunctiva firmly 3 times with a polyester-tipped swab (Fisher Scientific, MA, USA), rotating 120 degrees along the swab's axis each time to collect a sufficient epithelial specimen. The grader then placed the swab into a 2.0 ml nunc-tube, labeled the tube, and placed in a cooler bag with ice packs. Samples were then stored in the field in vaccine coolers (approximately 4º C) up to a maximum of approximately 15 days for remote districts far from the laboratory. When sample collection was complete, samples were transferred in vaccine coolers to the laboratory where they were stored in -20º C freezers until testing.

All laboratory testing was performed at the Amhara Public Health Institute in Bahir Dar, Ethiopia. Laboratory technicians were masked to the district of origin and trachoma status of each child providing the sample. Conjunctival swabs from each district were randomized and 5 samples were combined into each pool [4, 6, 20]. Pools were processed with the RealTi*m*e (Abbott Molecular Inc., Des Plaines, IL, USA) polymerase chain reaction (PCR) assay on the Abbott m2000 system between January 2015 and February 2016 to estimate the district prevalence of infection [4, 21]. The RealTi*m*e assay targets 2 highly conserved targets on the *Ct* plasmid and is highly sensitive (95.3%) and specific (99.9%) [22]. The time from sample collection to testing of pools ranged from 1,508 days to 119 days. All individual samples from the identified positive pools from 4 zones were processed again between January and December 2018 to provide individual level infection data. The time from sample collection to individual testing ranged from 1,148 days to 2,356 days. All individuals with a pooled negative result were assumed to be negative [4, 23–25].

In order to obtain a quantitative measure of *Ct* load, a standard set of elementary body (EB) titrations was created (Schachter laboratory, University of California San Francisco) [26]. The standards for these titrations were prepared from *Ct* grown in tissue culture. Forty-eight to 72-hour cultures were harvested, vortexed and purified by cycles of differential centrifugation. Reticulate bodies, being labile, were lost in this process. EB counts were then made by microscopy. Ten-fold dilutions of these preparations were then run through the m2000 to generate a calibration curve. For positive individual samples, the m2000 generated delta cycle result was converted to EB equivalent concentration based on this calibration curve. Strict laboratory quality control procedures were maintained throughout the project, including monthly amplicon testing for contamination, and external repeat testing at a laboratory in the United States as detailed previously [4].

### Data analysis

Infection was defined as present if *Ct* DNA was detected in a swab. District estimates of TF (with/without TI) and TI (with/without TF) were weighted using the inverse of probability of selection at each stage of selection and estimated using survey procedures accounting for the multi-level nature of the survey design (Stata svy commands; Stata Corporation, College Station TX, USA). Confidence intervals (CI) for district estimates were calculated accounting for clustering at the village and household level using Taylor linearization in Stata. District prevalence data were reported for the age group 1 to 9 years as is standard in the trachoma literature. All other data reported are for pre-school age children aged 1 to 5 years. District *Ct* prevalence was estimated from the pooled prevalence as the number of positive individuals most likely to have resulted in observed pooled results [4, 21].

Within the sample of pre-school aged children, clustering at the village and household level was accounted for using Taylor linearization via survey procedures in Stata. Since EB equivalent values were generated using a calibration curve, for analytic purposes, the relative EB values were of importance as opposed to the absolute values. Because of skewness in the EB load variable (kurtosis determined using “summary” command in Stata), the variable was transformed using the natural log among those positive for *Ct* infection. A chi-square (χ^2^) test was used to compare binary variables, and linear and logistic regression were used to examine relationships between dependent and independent variables with P < 0.05 considered statistically significant. A statistical test for trend (Ptrend) was conducted by including a categorical variable as an independent variable in the regression model. All analyses were conducted using Stata 13.1. Maps were created in ArcGIS 10.6 (ESRI, Redlands, CA).

## Results

**Fig 1:**
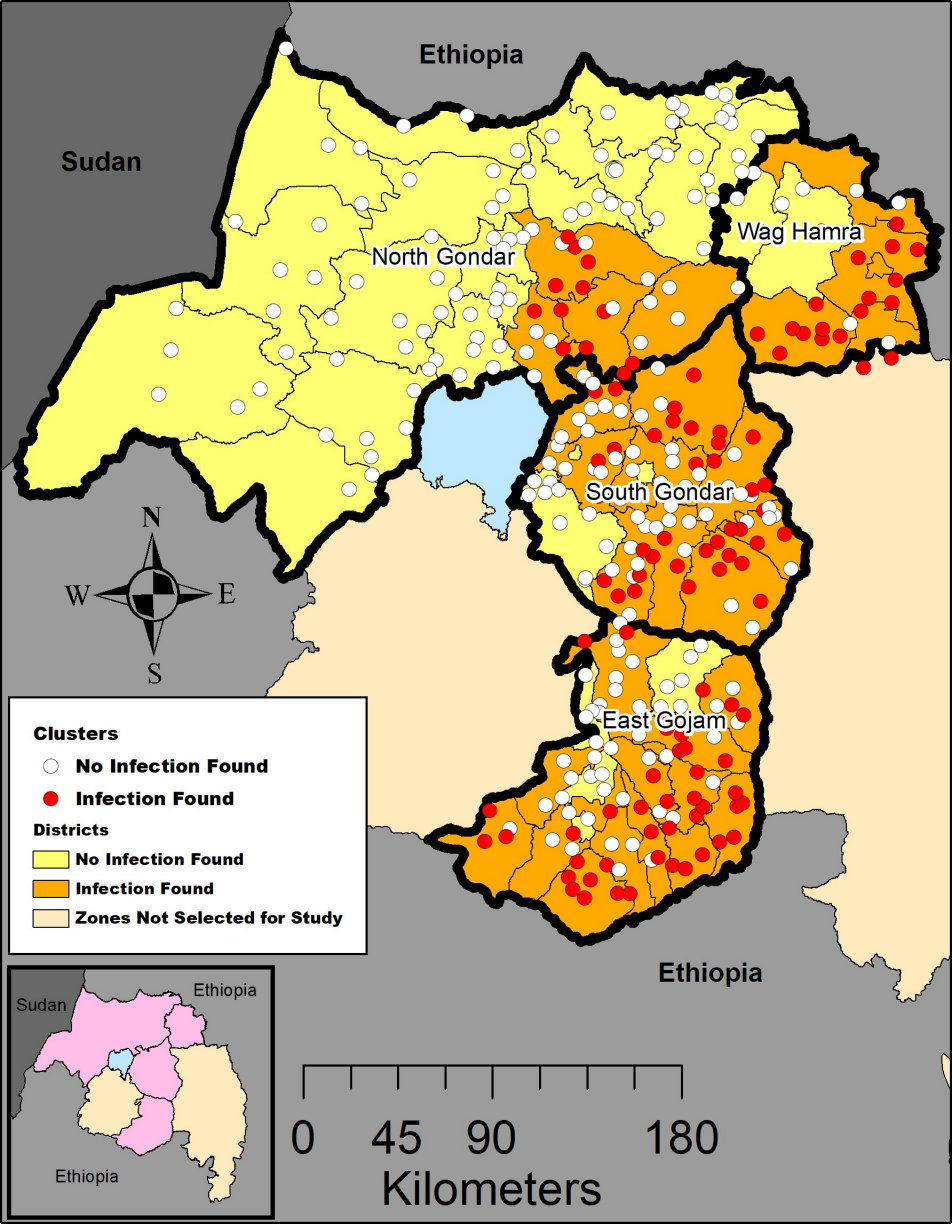
Location of surveyed clusters and clusters with positive sample among 4 administrative zones of Amhara, Ethiopia, 2011–2015. Map created in ArcGIS 10.6 (ESRI, Redlands, CA) using a customized shapefile originally sourced from the GADM database (gadm.org). Inset map: Amhara region, zones included in this study are shaded in pink.

Conjunctival swab samples were taken from a population-based sample of 7,441 children aged 1 to 5 years across 58 districts in 4 contiguous zones in Amhara (Fig 1; S1 Table). A total of 2,407 swabs were collected in 2011, 1,271 in 2012, 2,784 in 2013, 223 in 2014, and 756 in 2015 based on the year of the trachoma survey to measure the impact of approximately 5 years of the SAFE strategy. At least 1 positive swab was detected in 33 (56.9%) of the 58 districts within these zones. Among districts where infection was detected, the district prevalence of *Ct* infection among children 1 to 5 years ranged from 0.8% to 38.3% (S2 Fig). The district TF prevalence among children aged 1 to 9 years for these districts ranged from 17.8% (95% CI: 14.5%, 21.8%) in Farta district, South Gondar to 73.9% (95% CI: 49.0%, 89.3%) in Abergele district, Waghemra. Among districts without infection, the district TF prevalence ranged from 2.5% (95% CI: 1.1%, 5.7%) in Tach Armchiho, North Gondar to 33.9% (95% CI: 22.6%, 47.4%) in Ziqualla, Waghemra. Across the 33 districts with infection, 34.4% (115/334) of sampled clusters had individuals with infection (range of infected children per cluster 1–20 out of 25 swabbed). Most conjunctival swabs (5,414/7,441, 72.8%) were taken approximately 8 months (range = 7–9 months) after the last round of MDA. Twelve of the 342 (3.5%) positive pools did not result in a positive individual upon retesting. Those positive pools which contained negative individual samples upon retesting could be considered either low-level (≤5 delta cycles) positives (9/12, 75%), or medium level (5.0–8.0 delta cycles) positives (3/12, 25%).

**Fig 2:**
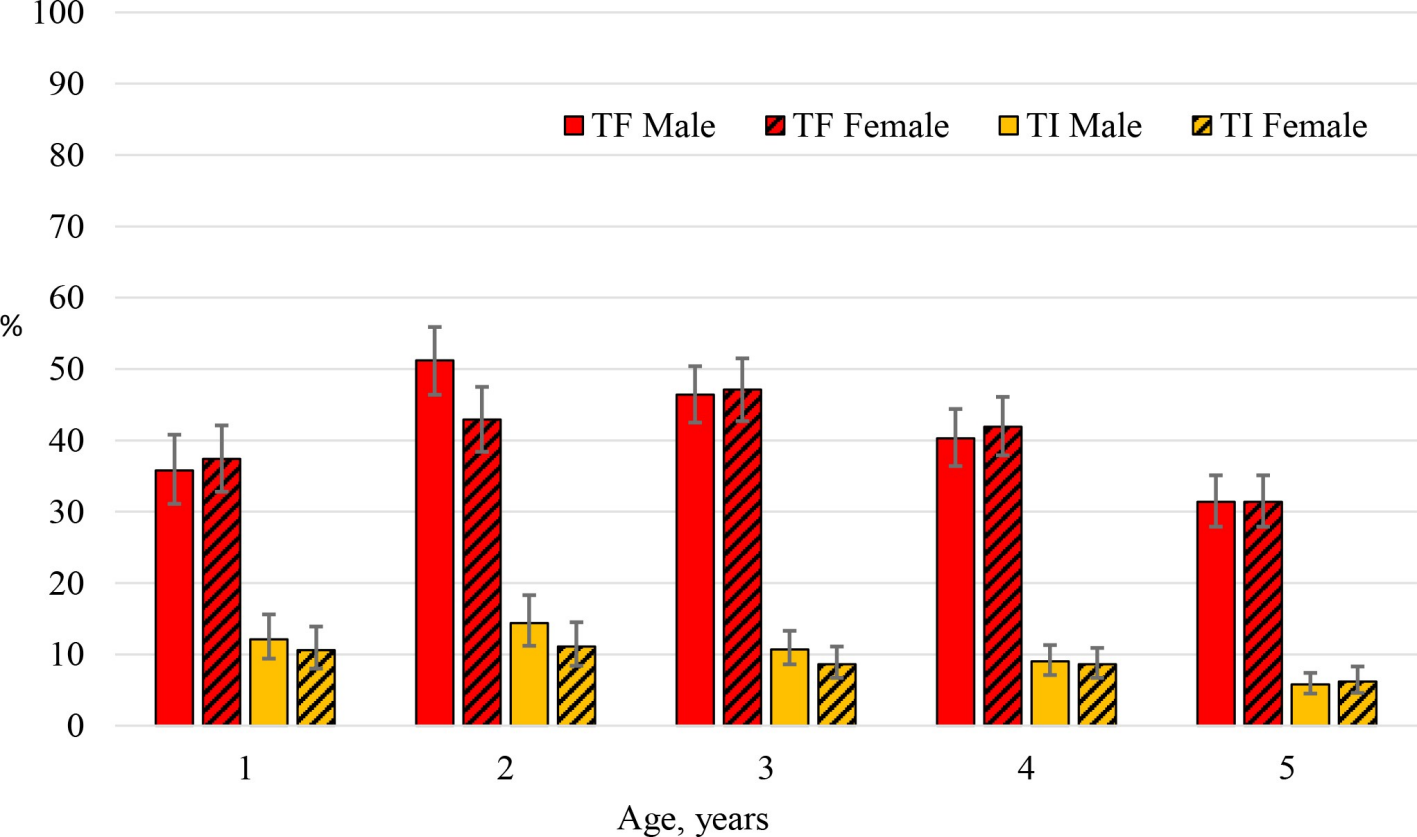
The age-sex specific prevalence and 95% confidence intervals of TF and TI among children aged 1 to 5 years, Amhara, Ethiopia, 2011–2015. Figure includes 7,254 children with complete age, sex and trachoma sign data.

The prevalence of TF among children aged 1 to 5 years in the sample was 39.9% (95% CI: 37.5%, 42.4%) and was highest in children 2 to 3 years of age (Fig 2). Male participants had a similar prevalence of TF when compared to female participants, 40.1% vs. 39.7% respectively (P = 0.71). The prevalence of TI in the sample was 9.2% (95% CI: 8.1%, 10.3%) with the highest prevalence observed among children aged 1 and 2 years of age. TI prevalence was similar for male (9.7%) and female (8.6%) participants (P = 0.11). A small percentage of participants (0.6%) showed signs of trachomatous scarring.

**Fig 3:**
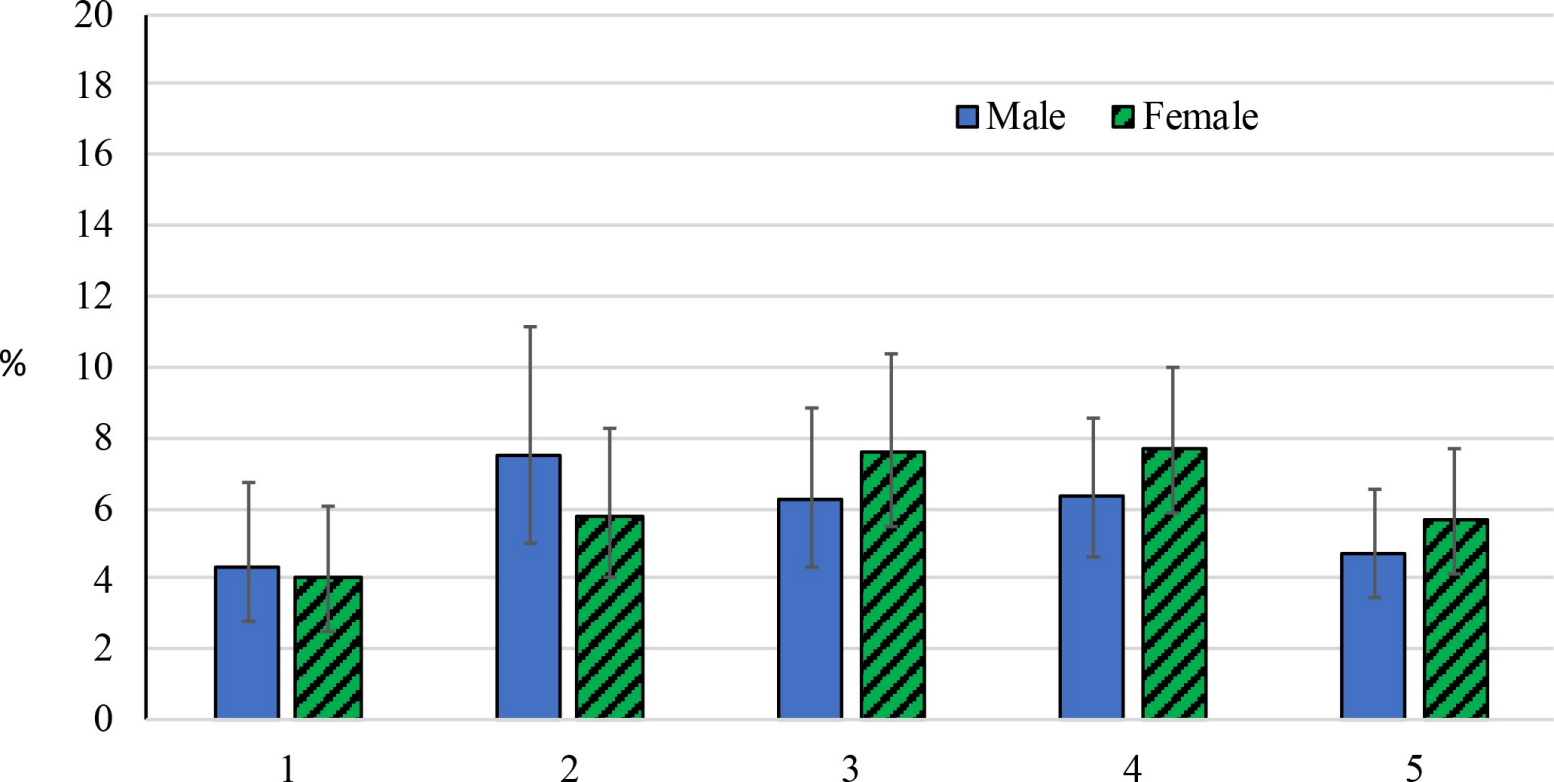
The age-sex specific prevalence of *Ct* infection and 95% confidence intervals among children aged 1 to 5 years, Amhara, Ethiopia, 2011–2015. Figure includes 7,285 children with complete age, sex and *Ct* infection data.

The prevalence of *Ct* infection among this cohort of children was 6.0% (95% CI: 5.0%, 7.2%). Among those positive for infection with available sex data (439/446), 232 (52.9%) were female. The *Ct* infection prevalence among females aged 1 to 5 years was 6.3% (95% CI: 5.1%, 7.7%), while the prevalence in males was 5.8% (95% CI: 4.7%, 7.1%), a non-statistically significant difference (P = 0.36). The prevalence of *Ct* infection was highest among children aged 2 to 4 years for both female and male participants (Fig 3). The highest prevalence was observed in males aged 2 years (7.5% [95% CI: 5.0%, 11.1%]) and in females aged 4 years (7.7% [95% CI: 5.9%, 10.0%]), and children aged 1 year had 9.6% of the infection prevalence among the cohort. Infection prevalence was highest in individuals with both TF and TI (31.4% [95% CI: 24.7%, 39.1%]), followed by individuals with TI only (12.3% [95% CI: 8.4%, 17.5%]) (Table 1). *Ct* infection did not increase with time since last MDA (7 months: 3.9% [95% CI: 2.2%, 6.9%], 8 months: 7.1% [95% CI: 5.8%, 8.6%], 9 months: 1.9% [95% CI: 0.5%, 6.4%]; P_trend_ = 0.74).

**Table 1:** *Ct* infection by active trachoma sign among children aged 1 to 5 years, Amhara, Ethiopia, 2011–2015.

Clinical Signs	Infection Status		
	Total individuals	Positive	Prevalence	95%CI
TF Only	2495	235	9.4%	7.7–11.4%
TI Only	261	32	12.3%	8.4–17.5%
TF and TI	404	127	31.4%	24.7–39.1%
No trachoma signs	4101	46	1.1%	0.8–1.6%
All participants	7441	446	6.0%	5.0–7.2%

**Fig 4:**
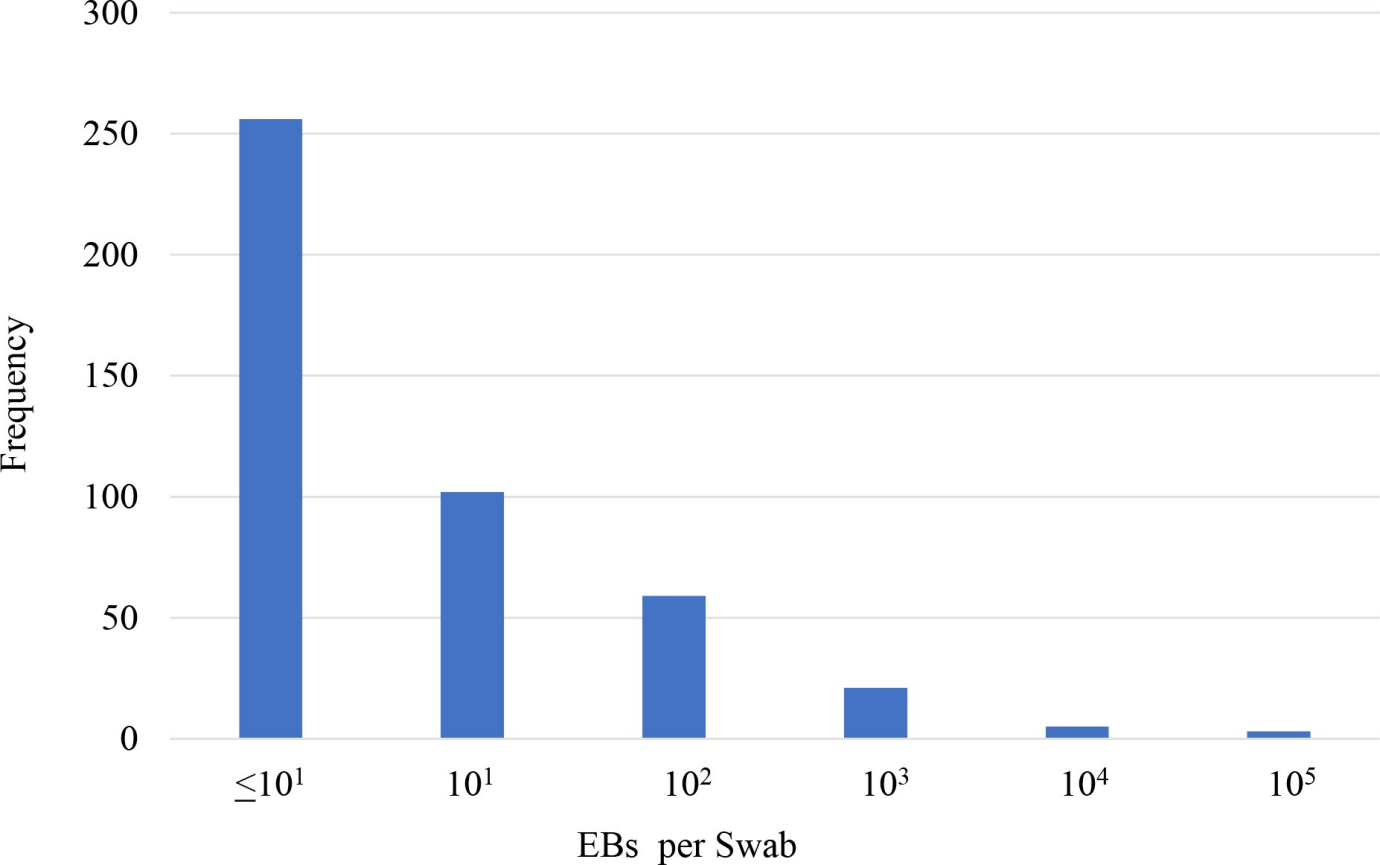
Distribution of elementary body count per swab among swabs positive for *Ct* infection, Amhara, Ethiopia, 2011–2015.

**Fig 5:**
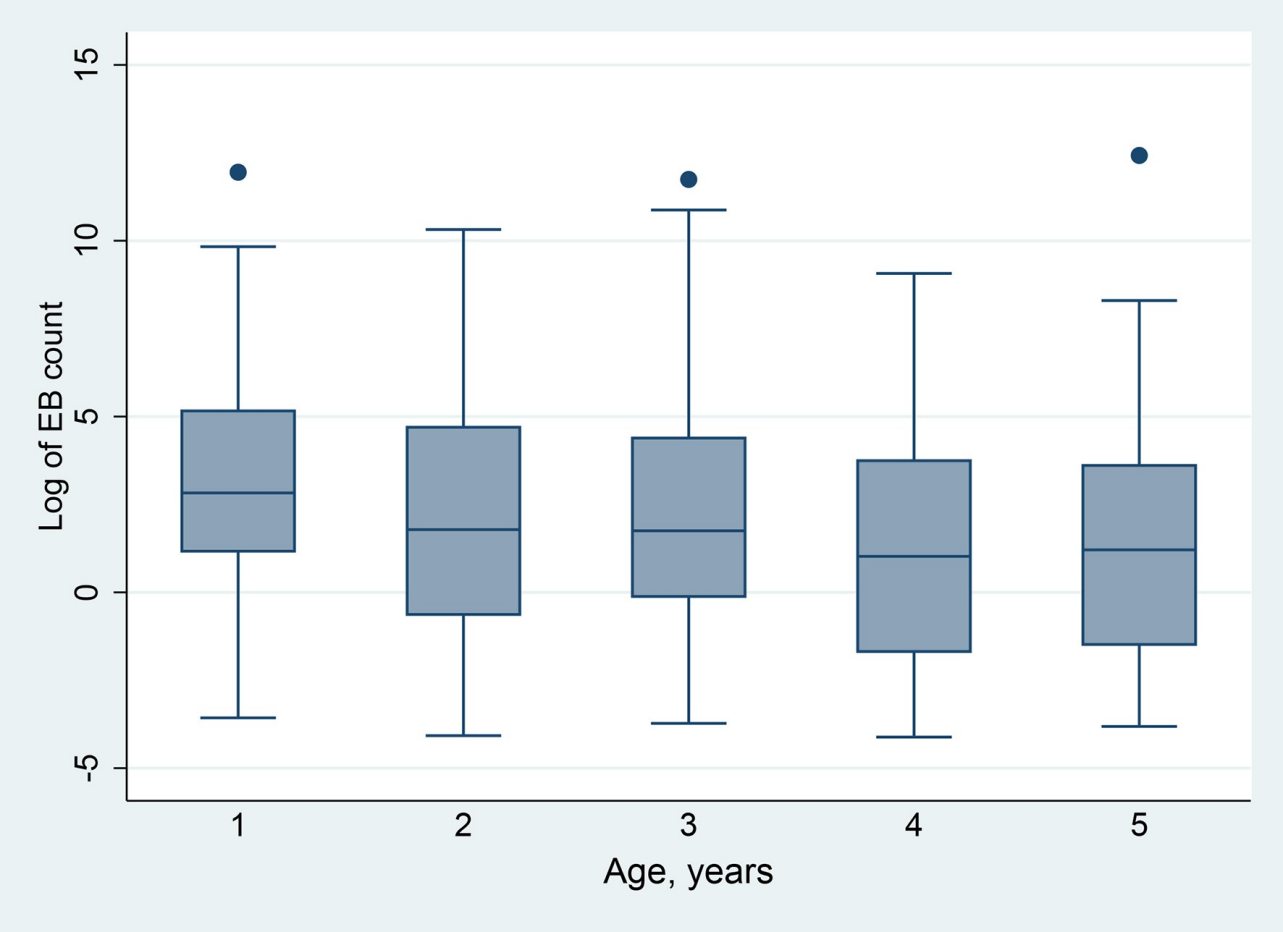
Boxplot of the distribution of elementary body count per swab (median, interquartile range, adjacent whiskers, and outliers) by age among swabs positive for *Ct* infection, Amhara, Ethiopia, 2011–2015. Figure includes 446 children with data on *Ct* infection and age.

**Fig 6:**
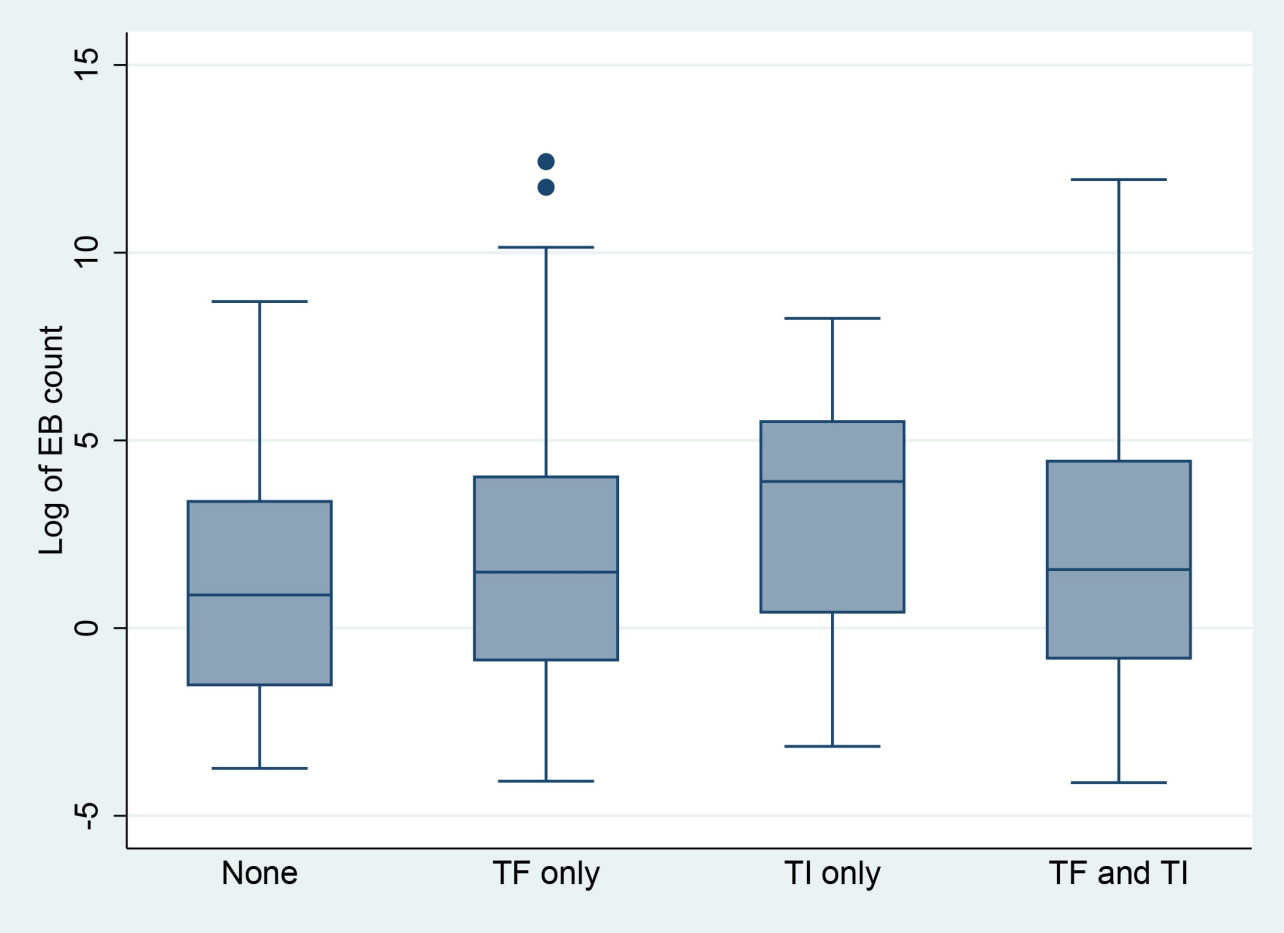
Boxplot of the distribution of elementary body count per swab (median, interquartile range, adjacent whiskers, and outliers) by active trachoma signs among swabs positive for *Ct* infection, Amhara, Ethiopia, 2011–2015. Figure includes 440 children with data on *Ct* infection and trachoma signs.

Among the 446 children positive for *Ct* infection, the range of *Ct* load was 0.16 EB to 248,935 EB, although the distribution was highly skewed (kurtosis = 180.69), with most infections consisting of ≤ 10 EBs (Fig 4). *Ct* load differed by age (β = -.41; P = 0.002), with the highest average loads observed in children aged 1 year (P = 0.01 vs. participants age aged 5 years) (Fig 5). Furthermore, nearly a quarter (23.5%) of the total chlamydial load in this sample was found in swabs taken from children aged 1 year. Mean *Ct* load was slightly higher in male participants (2.0 ln EBs [95% CI: 1.6 ln EBs, 2.5 ln EBs]) than female participants, (1.5 ln EBs [95% CI: 1.0 ln EBs, 1.9 ln EBs]) a difference that was not statistically significant (P = 0.11). Participants with TF only (1.6 ln EBs [95% CI: 1.2 ln EBs, 2.0 ln EBs]) had *Ct* loads higher than those participants without trachoma signs (1.0 ln EBs [95% CI: 0.1 ln EBs, 1.9 ln EBs]) but not statistically significantly so (P = 0.20), while those with TI only had the highest *Ct* loads (3.0 ln EBs [95% CI: 1.8 ln EBs, 4.1 ln EBs]), (P < 0.01) vs. those without signs (Fig 6).

## Discussion

Among pre-school aged children predominantly born during implementation of the SAFE strategy in trachoma hyperendemic Amhara, the prevalence of TF was 39.9%, while the prevalence of TI and *Ct* infection were 9.2% and 6.0% respectively. Post-SAFE interventions, infection prevalence was highest among participants aged 2 to 4 years, and a considerable proportion of overall *Ct* load was observed in children aged 1 year suggesting ongoing transmission and documenting the possibility of the youngest children maintaining a reservoir of infection in a community. Clearly SAFE interventions as implemented over a 5-year period in Amhara were not sufficient for reaching elimination as a public health problem in these hyperendemic districts. Using existing approaches, it is likely that many more years of SAFE will be required. The Trachoma Control Program in Amhara as well as those programs faced with hyperendemic trachoma should seek to increase investment in F and E interventions and should consider enhancements to the MDA strategy, such as alternative treatment regimens, to more swiftly eliminate trachoma as a public health problem in districts experiencing persistent trachoma.

Prior to the advent of the SAFE strategy, Amhara was hyperendemic for trachoma throughout the region, and there is evidence that the burden in Amhara was worse than that of other regions of Ethiopia [27, 28]. Among the 5 districts where the SAFE strategy was piloted in Amhara, the baseline prevalence of TF among children aged 1 to 9 years ranged from 49 to 90% between 2001 and 2003[14]. Between 2007 and 2010 the SAFE strategy was scaled up to reach all districts in the region, and interventions were administered for approximately 5 years until impact surveys were conducted between 2011 and 2015 [3]. Despite these interventions, the resulting prevalence of TF at impact survey was heterogeneous with 37% of the districts remaining hyperendemic [3]. Furthermore, *Ct* infection was found in all 10 zones with 1 zone having a prevalence of infection as high as 18.5% [4]. At the zonal level, TI and *Ct* prevalence point estimates in Amhara were similar in magnitude and were highly correlated [4]. This current report further demonstrated that among a cohort of young children post-MDA, TI prevalence more closely matched *Ct* infection prevalence and that individuals with TI had the highest *Ct* loads. Although TI is normally collected as part of trachoma impact surveys, it is not currently used as a programmatic indicator [29]. Given the correlation between TI and *Ct* infection at programmatically relevant enumeration units, and given the added costs associated with collection and testing of ocular swabs for infection, TI may be a cost-effective way for programs to better estimate the underlying infection levels within their populations. [30]. Further discussion on the role of TI in programmatic decision making is needed at the global level.

The infection results observed in the sample of children can be compared to previous results observed in other research settings. The distribution of *Ct* load was highly skewed in this population; most individuals had low load infections, while fewer individuals had high load infections. This same pattern was observed in Tanzania and Guinea Bissau in hyperendemic settings [9, 11, 12], as well as in the Gambia in a low prevalence setting [7]. The low loads detected here may represent resolving infections, and immunity may play an important role, whereby young children in highly endemic settings such as Amhara are able to better control their infections compared to children in less endemic settings [8]. High *Ct* loads were also observed among this sample of children 8 months after the last MDA. In general, individuals with high loads may be responsible for more disease transmission, and high loads may not be cleared by a single dose of azithromycin [8, 31]. Furthermore, children with high *Ct* loads prior to MDA have been shown to be more likely to be infected 6 months after MDA [8]. Programmatic targeting of core groups such as the youngest children or children harboring the highest *Ct* loads could be an important strategy for countries experiencing persistently high trachoma [32]. Several randomized trials which seek to test targeting strategies are planned for the region, and the cost and cost-effectiveness of these approaches should help determine their programmatic feasibility [32, 33]. Furthermore, longitudinal studies in Amhara would help to better elucidate the mechanisms of *Ct* infection transmission pre- and post-MDA.

Age-related patterns of *Ct* infection and infectious load observed in Amhara were similar to that in other hyperendemic settings. Previous studies have demonstrated, particularly after antibiotic distribution, that infection tends to cluster in the youngest children [12, 34, 35]. In Guinea Bissau, after 1 round of MDA, 59% of infection was found in children < 5 years [11], while in Tanzania, 87% of high load infections were found in children ≤ 5 years after MDA [9]. Among this cohort of pre-school children in Amhara, children aged 2 to 4 years had the highest prevalence of *Ct* infection, which further contributes to the body of evidence supporting an increased programmatic focus on this population subgroup [12, 34–36]. Our data also demonstrated that children aged 1 year had nearly a quarter of the sample load of infection approximately 8 months after MDA. A considerable proportion of these children were likely not yet born at the time of the MDA. The source of these infections, whether a sibling, a mother, or other household member could not be determined with the available data. The rest of these infected children were likely aged < 6 months at the time of treatment, and therefore would not have been eligible for MDA with azithromycin. These children would then represent a possible reservoir of infection that could hamper the effectiveness of MDA. Although we did not collect infection data on children aged < 1 year, other studies have demonstrated the presence of infection in infants [37], and have demonstrated that infants can harbor a considerable proportion of a communities *Ct* load [12]. Future work in hyperendemic regions should focus on whether children aged < 6 months, who are currently not treated with azithromycin, are potential reservoirs of infection.

Both commercial and “homebrew” nucleic acid amplification tests have been used in trachoma field studies to detect *Ct* infection. These tests are highly sensitive and specific, and some can be adapted to detect organism load in a specimen sample. Early methods used to determine *Ct* load were 2 step PCRs. Initially, a Roche Amplicor test was performed followed by a retest of positives with a real time quantitative PCR [12]. However, only 85% of the positives could be quantified as the real time assay was a less sensitive test than Amplicor. More recently, droplet digital PCR, a test with 73% sensitivity and 99% specificity compared to Amplicor, has been used to determine *Ct* copy number (load) [38].^.^The RealTi*m*e is a PCR assay that detects *Ct* DNA using the automated Abbott *m*2000 s system. It has a performance profile comparable to the Aptima Combo 2 [39], and the RealTi*m*e results are quantitative for *Ct* load. Because the m2000 system also allows a high throughput of samples, especially when pooling, large numbers of samples from various research projects, including routine monitoring and clinical trial research, have been assayed within the Amhara region itself [4, 20]. Previously published quality control data from this laboratory has demonstrated that high quality, replicable infection assays can be performed in country as part of a trachoma control program. The use of infection data in programmatic decisions warrants further discussion, particularly given the consistent disconnect between TF and actual *Ct* infection.

The results of this study should be considered in light of several limitations. The study was cross-sectional, and data were not available on infection prevalence prior to MDA. This limited the ability to know whether the infections detected were new infections since the last MDA or were infections that had not resolved in spite of the MDA. It is also possible that existing infection, particularly the high load infections, were those children missed by the MDA program. Although it is difficult to measure MDA coverage accurately in large scale programs, prior work has shown that administrative coverage in these zones is typically high and that self-reported coverage within the region is close to or above 80% [13, 40, 41]. This study swabbed individuals aged 1 to 5 years, restricting the interpretation to pre-school age children, although studies consistently show this age range to be the most epidemiologically relevant [12, 34, 35]. The time between sample collection and freezing at -20º C, the length of time a sample was frozen, or the number of freeze/thaw episodes experienced by a sample may have contributed to the low loads of *Ct* DNA detected in this study. However, in general bacterial DNA has been shown to be stable under various conditions, and cold chain was maintained throughout this study [42, 43]. Due to the inherent variability in quantifying *Ct* DNA in the laboratory, the absolute values of *Ct* loads presented here as EB equivalents, and used for within-study comparisons, should not be compared with other studies which use different methods.

Approximately 8 months after the 5^th^ round of MDA in Amhara, pre-school age children born during SAFE implementation still harbored considerable infection with ocular *Ct*. In this hyperendemic setting, infection prevalence was highest among children aged 2 to 4 years, and infectious load burden was highest among children aged 1 year. These young children likely contributed in meaningful ways towards the persistent trachoma observed in some districts. Treatment regimens focused on the youngest children or children harboring the highest *Ct* infectious loads should be explored to help countries experiencing persistent active trachoma reach elimination as a public health problem faster.

## Supporting information

S1 Fig Administrative mass drug administration coverage over time among 58 districts in North Gondar, South Gondar, East Gojam and Waghemra zones, Amhara, Ethiopia. (DOCX)
(DOCX)

S2 Fig **District level prevalence of a) *Chlamydia trachomatis* infection among children aged 1 to 5 years and b) trachomatous inflammation-follicular among children aged 1 to 9 years, 2011–2015, Amhara, Ethiopia.** Map created in ArcGIS 10.6 (ESRI, Redlands, CA) using a customized shapefile originally sourced from the GADM database (gadm.org).
(DOCX)

S1 Table Characteristics of children aged 1 to 5 years from the population-based sample (n = 7,441) and from children with a positive ocular swab for *Ct* infection (n = 446), Amhara, Ethiopia, 2011–2015. (DOCX)
