# Supplementary material for: Ocular Chlamydia trachomatis infection and infectious load among pre-school aged children within trachoma hyperendemic districts receiving the SAFE strategy, Amhara region, Ethiopia
Source: PLoS Negl Trop Dis. 2020 May 18;14(5):e0008226. doi: 10.1371/journal.pntd.0008226 (PMC7259799; doi:10.1371/journal.pntd.0008226)

Supplemental Figure 1. Annual administrative mass drug administration coverage over time among 58 districts in North Gondar, South Gondar, East Gojam and Waghemra zones, Amhara, Ethiopia.


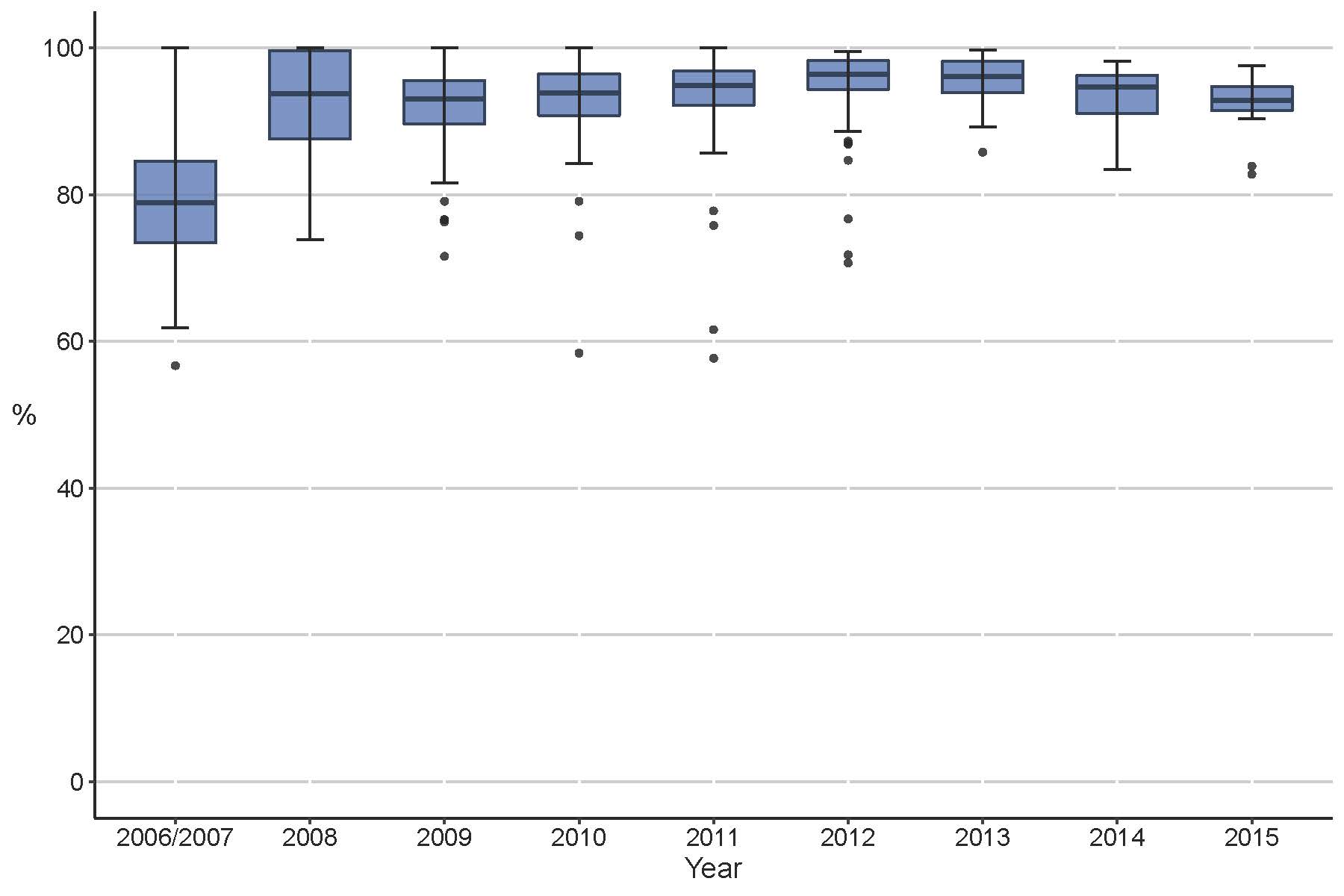

Supplement: S1 Fig — (DOCX) (DOCX) [file pntd.0008226.s001.docx]
