# Supplementary material for: Ocular Chlamydia trachomatis infection and infectious load among pre-school aged children within trachoma hyperendemic districts receiving the SAFE strategy, Amhara region, Ethiopia
Source: PLoS Negl Trop Dis. 2020 May 18;14(5):e0008226. doi: 10.1371/journal.pntd.0008226 (PMC7259799; doi:10.1371/journal.pntd.0008226)

Supplemental Figure 2. District level prevalence of a) *Chlamydia trachomatis* infection among children aged 1 to 5 years and b) trachomatous inflammation-follicular among children aged 1 to 9 years, 2011-2015, Amhara, Ethiopia. Map created in ArcGIS 10.6 (ESRI, Redlands, CA) using a customized shapefile originally sourced from the GADM database (gadm.org).


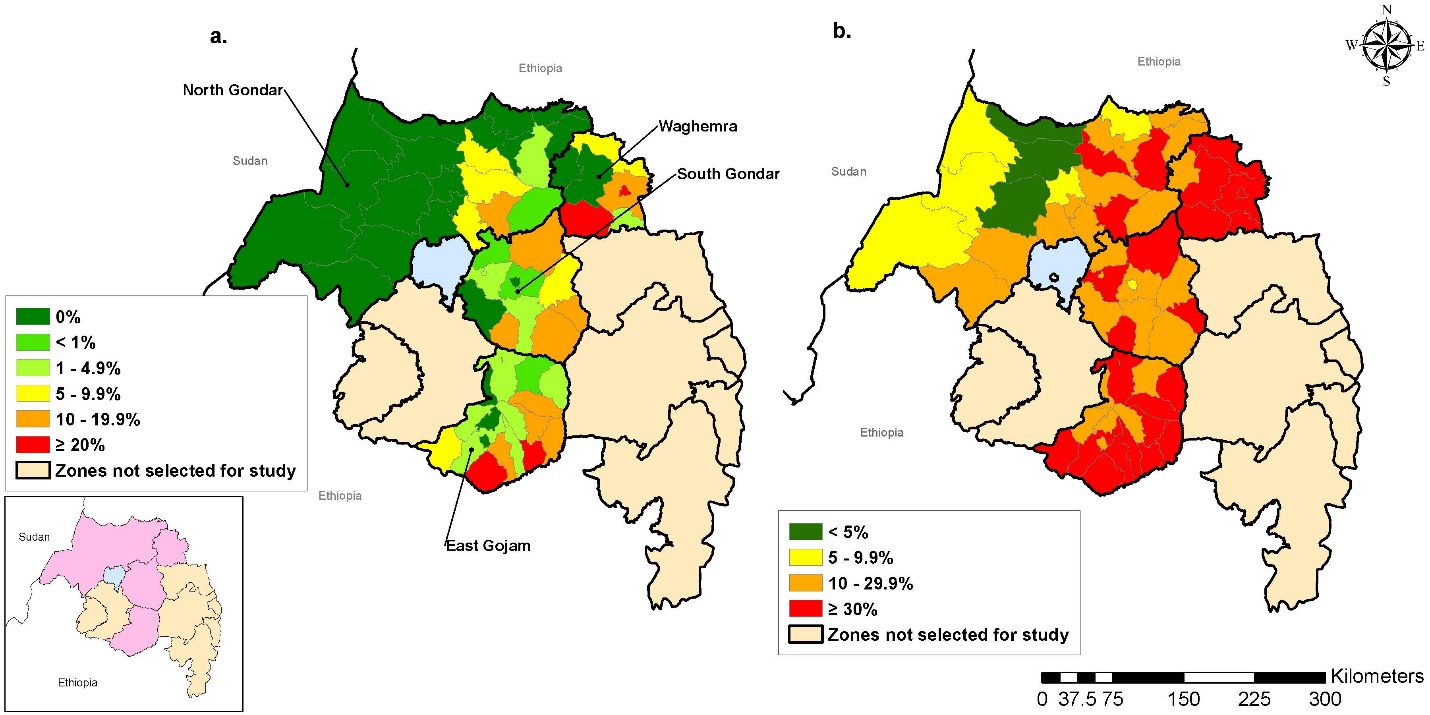

Supplement: S2 Fig — District level prevalence of a) Chlamydia trachomatis infection among children aged 1 to 5 years and b) trachomatous inflammation-follicular among children aged 1 to 9 years, 2011–2015, Amhara, Ethiopia. Map created in ArcGIS 10.6 (ESRI, Redlands, CA) using a customized shapefile originally sourced from the GADM database (gadm.org). (DOCX) [file pntd.0008226.s002.docx]
