# Supplementary material for: Ocular Chlamydia trachomatis infection and infectious load among pre-school aged children within trachoma hyperendemic districts receiving the SAFE strategy, Amhara region, Ethiopia
Source: PLoS Negl Trop Dis. 2020 May 18;14(5):e0008226. doi: 10.1371/journal.pntd.0008226 (PMC7259799; doi:10.1371/journal.pntd.0008226)
Supplement: S1 Table — (DOCX) [file pntd.0008226.s003.docx]

**Supplementary Table 1*.*** Characteristics of children aged 1 to 5 years from the population-based sample (n=7,441) and from children with a positive ocular swab for *Ct* infection (n=446), Amhara, Ethiopia, 2011-2015

|  | Total Swab Sample | Positive Swab |
| --- | --- | --- |
| Characteristic | N (%) ^a^ | N (%) ^b^ |
| Sex |  |  |
| Male | 3,594 (49.3) | 207 (47.2) |
| Female | 3,691 (50.7) | 232 (52.9) |
| Age, years |  |  |
| 1 | 1,038 (14.0) | 43 (9.6) |
| 2 | 1,150 (15.5) | 76 (17.0) |
| 3 | 1,529 (20.6) | 106 (23.8) |
| 4 | 1,687 (22.7) | 116 (26.0) |
| 5 | 2,030 (27.3) | 105 (23.5) |
| TF Present | 2,899 (39.9) | 362 (82.3) |
| TI Present | 665 (9.2) | 159 (36.1) |
| Zone |  |  |
| East Gojam | 1,825 (24.5) | 171 (38.3) |
| North Gondar | 2,622 (35.2) | 63 (14.1) |
| South Gondar | 2,407 (32.4) | 125 (28.0) |
| Waghemra | 587 (7.9) | 87 (19.5) |

TF=trachomatous inflammation-follicular; TI=trachomatous inflammation-intense

^a^ Missing data for total sample: 156 for sex, 7 for age, 644 for clean face, 180 for TF and TI

^b^ Missing data for positive swab sample: 7 for sex, 33 for clean face, 6 for TF and TI
